# ctDNA as a prognostic biomarker in resectable CLM: Systematic review and meta-analysis

**DOI:** 10.1515/biol-2022-0615

**Published:** 2023-05-19

**Authors:** Da Wang, Penglai Zhao, Tingting Lu, Jingyao Ren, Lihui Zhu, Xiaoyong Han, Guangming Zhang, Xiaohua Dong, Haizhong Ma, Miao Yu, Hui Cai

**Affiliations:** School of Medicine Jiangsu University, Zhenjiang, 212000, China; General Surgery Clinical Medical Center, Gansu Provincial Hospital, Lanzhou, 730000, China; Key Laboratory of Molecular Diagnostics and Precision Medicine for Surgical Oncology in Gansu Province, Gansu Provincial Hospital, Lanzhou, 730000, China; The First Clinical Medical College, Gansu University of Chinese Medicine, Lanzhou, 730000, China; Institution of Clinical Research and Evidence Based Medicine, Gansu Provincial Hospital, Lanzhou, 730000, China; First Clinical College of Medicine, Lanzhou University, Lanzhou, 730000, China; School of Clinical Medicine Ning Xia Medical University, Yinchuan, Ning Xia, 750004, China

**Keywords:** ctDNA, colorectal cancer liver metastasis, meta-analysis, prognostic biomarker

## Abstract

Cell-free circulating tumor DNA (ctDNA) is synthesized by tumor cells, including metastatic tumors, and circulates in the bloodstream. Evidence suggests that ctDNA is a potential predictive and prognostic biomarker for colorectal cancer (CRC), but its predictive efficacy in detecting CRC liver metastasis (CLM) remains unclear. Additionally, its utility in the clinical setting needs further investigation. We conducted a meta-analysis to determine the utility of ctDNA as a biomarker for predicting the prognosis of CLM and investigate the relationship between CLM and ctDNA positivity. A literature search was performed in electronic databases to identify relevant studies published up to March 19, 2022. We retrieved data on overall survival (OS), disease-free survival (DFS), and recurrence-free survival (RFS) for both ctDNA-positive and ctDNA-negative colorectal liver metastasis (CLM) patients from the selected articles. Hazard ratios (HRs) were also calculated for these survival outcomes analysis was also performed. The stability of the combined meta-analysis was verified by sensitivity analysis and publication bias evaluation. Ten trials were included, and 615 patients were evaluated. In patients with CLM, pooled HRs revealed a substantial link between ctDNA positivity and RFS/DFS. Subgroup analysis revealed that ctDNA had a prospective detection value. Sensitivity analysis and publication bias evaluation indicated stable results. Although the results on pooled HR for OS suggested that ctDNA-positive patients had a shorter survival time, their pooled HRs had a relatively evident heterogeneity, and sensitivity analysis and publication bias evaluation indicated that pooled HRs were extremely unstable. In conclusion, our results demonstrate that ctDNA appears to be a prognostic biomarker for resectable CLM patients.

## 1 Introduction

Colorectal cancer (CRC) ranks third in incidence rates and second in mortality rates worldwide [1]. Approximately 25% of the CRC patients are clinically diagnosed with liver metastases at the time of CRC diagnosis, nearly 50% of CRC patients develop liver metastases during the disease course, and approximately 30–50% of CRC patients experience recurrent liver metastases after radical resection. Liver metastasis is a leading cause of mortality in CRC [2,3,4]. However, owing to the rapid progression of CRC metastases and drug resistance, currently available treatment strategies surgery, radiotherapy, and chemotherapy for patients with CRC liver metastasis (CLM) have shown unsatisfactory results [5,6]. Early diagnosis of tumor progression and metastasis remains the most effective strategy for improving patient prognosis [7].

Imaging (computed tomography [CT]) and serum tumor markers (carcinoembryonic antigen [CEA] and carbohydrate antigen 19-9 [CA19-9]) are the currently available approaches for detecting disease progression. CEA is used as a marker to diagnose rectal cancer, and CA19-9 is used as a marker for pancreatic and other cancers, as these markers are specifically expressed in these cancers [8,9]. CT is ineffective in detecting minimal residual disease but has poor sensitivity and specificity [10]. With further advancements in imaging technology, particularly the introduction of radiomics and radiogenomics, the detection of CLMs and postoperative recurrence has improved significantly [11]. However, compared with serum tumor markers, imaging does not reliably predict survival outcomes. The most frequently used serum tumor markers for rectal cancer detection are CEA and CA 19-9, although their use is restricted owing to poor sensitivity and specificity [10,12]. There is an urgent need for identifying tools with high reliability, sensitivity, and specificity for the identification of postoperative tumor recurrence. Therefore, new approaches must be developed to enhance diagnosis, evaluate patient-specific therapy, and perform disease monitoring.

The field of liquid biopsy for tumors has rapidly expanded owing to its safety and minimally invasive nature, particularly with the emergence of cell-free circulating tumor DNA (ctDNA). ctDNA is synthesized in tumor cells and released into the bloodstream after necrosis, apoptosis, and secretion [12,13,14]. Metastatic tumors can enter other organs and tissues through lymphatic vessels or blood vessels and release ctDNA at these locations [15]. The primary techniques for detecting ctDNA are single- or multi-point detection centered on real-time polymerase chain reaction (PCR) and targeted sequencing [13,16]. Liquid biopsy-derived ctDNAs have the potential to significantly enhance diagnosis, prognosis, medication response prediction, and therapy response monitoring [12]. ctDNA can be used to evaluate the dynamic features of CRC [17,18], monitor therapy response, stratify treatment strategies [19,20,21,22,23], and predict survival [24,25]. A meta-analysis of non-metastatic CRC has shown that ctDNA is a potential biomarker for postoperative tumor recurrence [12].

Although ctDNA is a potentially useful predictive and prognostic biomarker for CRC, its role as a marker for the prognosis and recurrence of CLM has not been fully established, and no consensus exists on ways to use ctDNA most effectively in the clinical setting [26,27]. Therefore, we conducted a meta-analysis to determine the utility of ctDNA as a biomarker for predicting the prognosis and recurrence of CLM and investigate the relationship between CLM and ctDNA positivity.

## 2 Methods

At study initiation, we registered the study protocol for this literature review and meta-analysis in PROSPERO (No: CRD42022316060) [28].

### 2.1 Search strategy

A literature search was performed in electronic databases such as PubMed, Medline, Cochrane Library, Science Core Collection Network, and Embase from database inception to March 19, 2022. The search strategy was based on MeSH terms and free terms, and the search keywords included “colon cancer,” “rectal cancer,” “liver metastasis,” “cell-free DNA,” and “circulating tumor NDA.” Appendix 1 presents a detailed search strategy.

### 2.2 Inclusion and exclusion criteria

The criteria for study selection were as follows: (1) use of histopathology or cytology for the diagnosis of colon or rectal cancer; (2) use of radiography or histopathology to confirm liver metastasis; (3) evaluation of survival outcomes associated with ctDNA, including overall survival (OS), disease-free survival (DFS), and recurrence-free survival (RFS); and (4) study type being limited to a cohort study.

The exclusion criteria were as follows: (1) article type being letters, case reports, expert opinions, and reviews; (2) duplicate publications; (3) animal experiments; and (4) studies lacking original data.

### 2.3 Data extraction

Data extraction was performed individually by two researchers (Da Wang and Jingyao Ren), and when there was disagreement, a third researcher established consensus (Penglai Zhao). We independently extracted and reviewed the data of the following parameters from each eligible study: first author, country (first author), year of publication, number of patients enrolled, sample source, assay, marker used, time of sample measurement, treatment modality, duration of follow-up, outcomes, and results of quality assessment.

### 2.4 Quality assessment

The Newcastle–Ottawa Scale (NOS) was used to evaluate the quality of all included studies, and this assessment was performed independently by two researchers (Da Wang and Penglai Zhao). NOS values were assigned on a scale of 0–9, with scores higher than 6 indicating high-quality investigations [29].

### 2.5 Statistical analysis

For data analysis, we used Stata, version 12. Combined data of hazard ratios (HRs) and 95% confidence intervals (CIs) were extracted from the selected studies. The chi-square *Q* test and *I* ^2^ statistics were used to determine the heterogeneity of eligible studies. When *I* ^2^ > 50% or *p* < 0.05, we considered heterogeneity to be significant and adopted a random-effects model. In the other cases, a fixed-effects model was used. Publication bias was assessed through funnel plot asymmetry using Egger’s and Begg’s tests.

To gain insights into the prognostic significance of the sampling time points, we separately evaluated the HR of ctDNA at different sampling time points. Subgroup analysis was performed based on the characteristics of the literature, including detection method applied, marker used, sample type, statistical method adopted, and statistical tests used. The study followed the Preferred Reporting Items for Systematic Reviews and Meta-Analysis guidelines and the Meta-Analysis of Observational Studies in Epidemiology guidelines.

## 3 Results

### 3.1 Study selection

**Figure 1:**
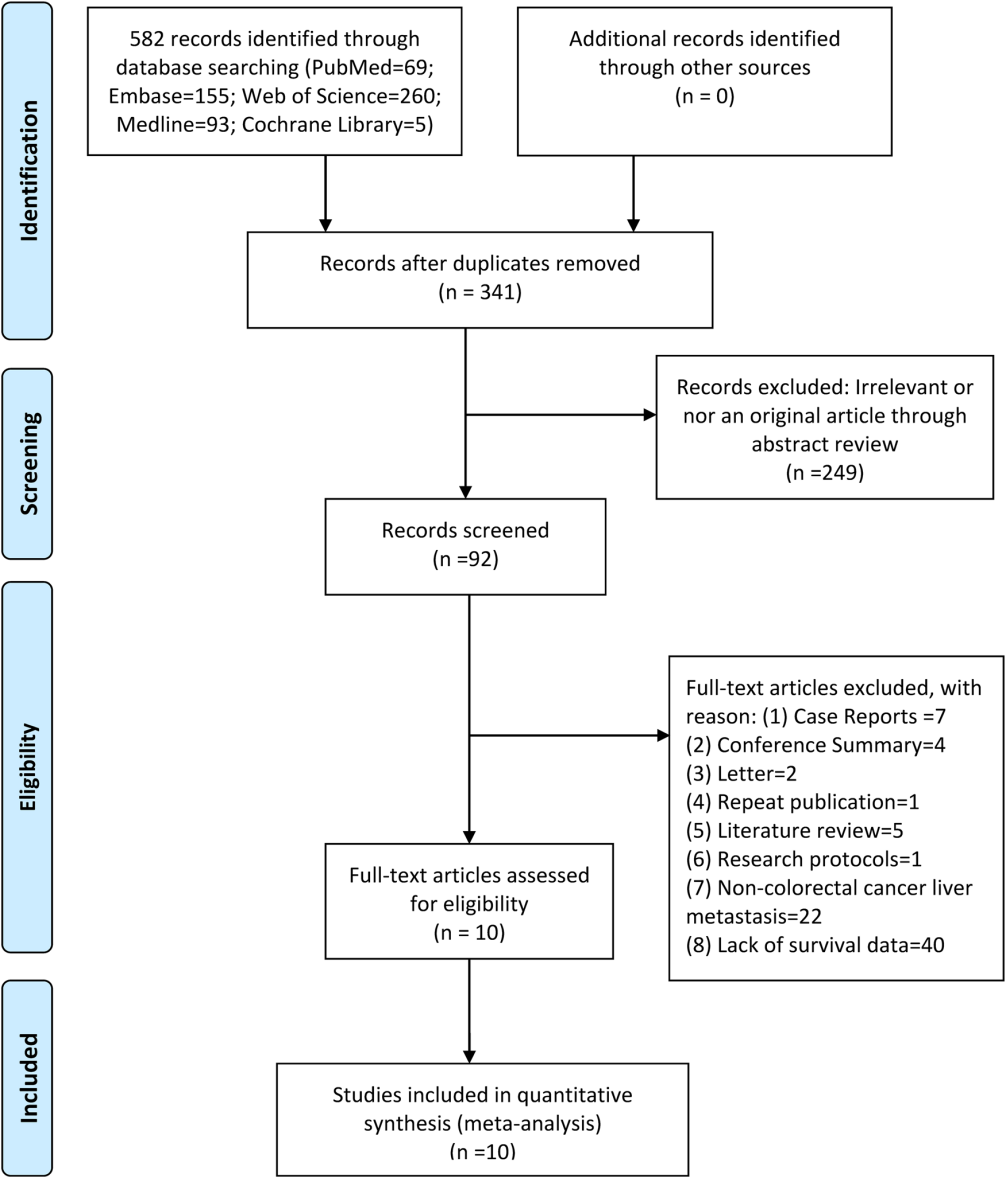
Flow diagram of literature search and study selection.

The literature search in the electronic databases yielded 582 articles, which were selected on consensus. Following a careful review of the titles and abstracts, 390 irrelevant papers and duplicate publications were excluded, of which 92 were selected for additional screening based on the full text. Eighty-one studies were omitted because ctDNA was not directly associated with CLM, survival analysis was not performed, or the reported data were inadequate. Finally, 11 articles fulfilled the inclusion and exclusion criteria [10,30,31,32,33,34,35,36,37,38] (Figure 1).

### 3.2 Characteristics of eligible studies and quality assessment

The major clinical features of the 11 eligible studies are presented in Table 1. This meta-analysis included 11 trials involving 615 patients with CLM, and sample sizes of the trials ranged from 18 to 115 individuals [10,30,31,32,33,34,35,36,37,38]. During 2017–2022, the selected studies were published in seven countries or regions. Safe sequencing system (SafeSeqS, *n* = 1), droplet digital PCR (ddPCR, *n* = 6), next-generation sequencing (NGS, *n* = 2), and Guardant360 (*n* = 1) were the four analytical methods used in the selected trials for detecting cell-free ctDNA. Moreover, the blood samples used for detecting ctDNA were obtained from patients with CRC at various phases of treatment, including baseline (*n* = 8), post-treatment (*n* = 8), and treatment completion (*n* = 3). One study used whole blood for detection, whereas the other studies used only plasma samples. The 11 studies included in this meta-analysis reported survival prognoses through various measures, with seven reporting RFS, three reporting OS, one reporting progression-free survival, and two reporting DFS. NOS was used to determine the quality of each study, and studies with a score of ≥6 were considered to have high quality.

**Table 1:** Study characteristics of included studies

Study	Country	Cohort study design	Detection methods	Markers	Marker origin	Sampling time	Patient No.	Cut off	Endpoints	HR	Multivariate/univariate analysis	NOS
Polivka et al., 2020 [33]	Czech Republic	Retrospective	ddPCR	KRAS	Plasma	Baseline	71	Median	OS, DFS	Reported in text	Multivariate	6
Reinert et al., 2022 [34]	Denmark	Prospective	ddPCR	APC, BRAF, KRAS, NRAS, PIK3CA, TP53	Plasma	Baseline/Post-treatment	115	Median	RFS	Reported in text	Univariate	8
Tie et al., 2021 [36]	Australia	Prospective	Safe-Sequencing	SMAD4, TP53, AKT1, APC, BRAF, CTNNB1, ERBB3, FBXW7, HRAS, KRAS, NRAS, PIK3CA, PPP2R1A, RNF43, POLE	Plasma	Baseline/Post-treatment/End of treatment	61	Median	OS, RFS	Reported in text	Univariate	9
Kobayashi et al., 2021 [30]	Japan	Retrospective	Guardant360	74 genes	Plasma	Baseline	40	Median	RFS	Reported in text	Univariate	8
Narayan et al., 2019 [31]	American	Prospective	NGS	APC, BRAF, EGFR, ERBB2, KRAS, NRAS, PIK3CA, SMAD4, TP53	Whole blood	Post-treatment	59	Median	DFS	Reported in text	Univariate	7
Øgaard et al., 2022 [32]	Denmark	Prospective	ddPCR	C9orf50, CLIP4, KCNQ5	Plasma	Baseline/Post-treatment/End of treatment	96	Median	RFS	Reported in text	Univariate	6
Wang et al., 2021 [37]	China	Prospective	NGS	The exome regions of 451 genes	Plasma	Baseline/Post-treatment/End of treatment	91	Median	RFS	Reported in text	Univariate	6
Bolhuis et al., 2021 [10]	Netherlands	Prospective	ddPCR	KRAS	Plasma	Post-treatment	23	Median	RFS	Reported in text	Multivariate	6
Bidard et al., 2019 [38]	France	Prospective	ddPCR	KRAS	Plasma	Post-treatment	21	Median	OS	Reported in text	Univariate	6
Scholer et al., 2017 [35]	Denmark	Prospective	ddPCR	KRAS	Plasma	Baseline	18	Median	OS, RFS	Reported in text	Univariate	5

ddPCR, droplet digital PCR; Safe-SeqS, Safe-Sequencing System; NGS, next-generation sequencing; OS, overall survival; PFS, progression-free survival; DFS, disease-free survival; RFS, recurrence-free survival; NOS, Newcastle–Ottawa Scale.

### 3.3 Effect of ctDNA detection on the prognosis of resectable CLM

**Figure 2:**
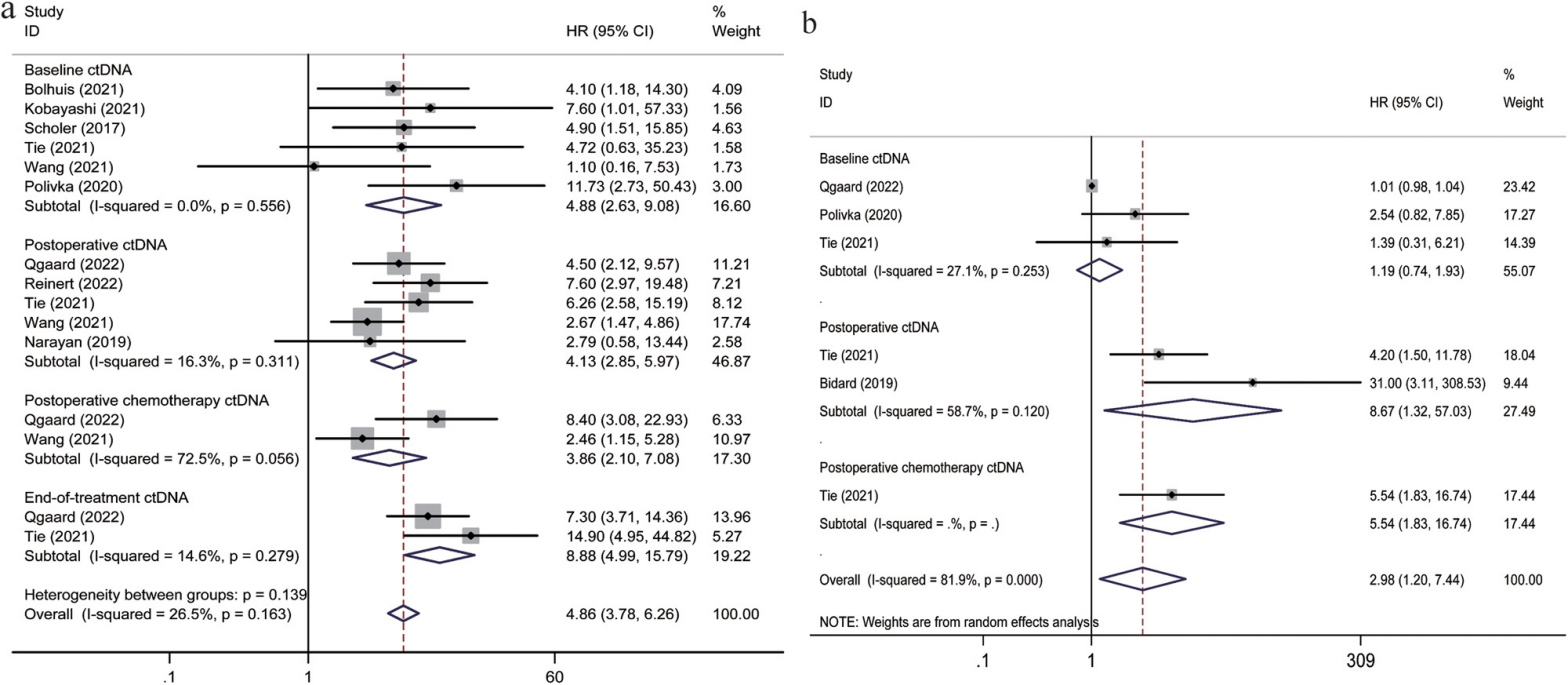
(a) Forest plot demonstrating the relationships between ctDNA and RFS/DFS. (b) Forest plot demonstrating the relationship between ctDNA and OS.

Figure 2 shows pooled date of the random-effects models for RFS/DFS and OS. Fifteen studies reported that ctDNA was related to RFS/DFS (six studies used baseline samples, five used postoperative samples, two used postoperative chemotherapy samples, and two used end-of-treatment samples). The risk of tumor recurrence indicated the tendency of increase in the ctDNA-positive group than in the ctDNA-negative group, regardless of the time point of sample collection (at baseline, postoperative, postoperative chemotherapy, and end of treatment) (detectable baseline ctDNA, pooled HR = 4.88, 95% CI: 2.63–9.08, *p* < 0.001; detectable postoperative ctDNA, pooled HR = 4.13, 95% CI 2.85–5.97, *p* < 0.001; detectable postoperative chemotherapy ctDNA, pooled HR = 3.86, 95% CI 2.10–7.08, *p* = 0.016; detectable end-of-treatment ctDNA, pooled HR = 8.88, 95% CI 4.99–15.79, *p* < 0.001; overall, pooled HR = 4.86, 95% CI 3.78–6.26, *p* < 0.001). Correlation studies between ctDNA detection and RFS/DFS revealed considerable heterogeneity (*I* ^2^ = 25.6%; *p* = 0.163). Therefore, we used a fixed-effects model (Figure 2a).

There was a higher degree of heterogeneity in OS for ctDNA positivity than in OS for ctDNA negativity (*I* ^2^ = 81.9%; *p* < 0.001); thus, so we used a random-effects model in the final analysis. As shown in Figure 2b, the OS time was lower in ctDNA-positive patients than in ctDNA-negative patients (detectable baseline ctDNA, pooled HR = 1.19, 95% CI: 0.74–1.93, *p* = 0.477; detectable postoperative ctDNA, pooled HR = 8.67, 95% CI 1.32–57.03, *p* = 0.025; detectable postoperative chemotherapy ctDNA, pooled HR = 5.54, 95% CI 1.83–16.74, *p* = 0.002; detectable end-of-treatment ctDNA, pooled HR = 8.88, 95% CI 4.99–15.79; overall, pooled HR = 2.98, 95% CI 1.20–7.44, *p* = 0.019).

### 3.4 Subgroup analysis

Based on the previous heterogeneity results, subgroup analyses were performed using a fixed-effects model for DFS/RFS (Table 2) and a random-effects model for OS (Table 3).

**Table 2:** Subgroup analysis: effect of ctDNA on RFS/DFS according to the various subgroups is presented

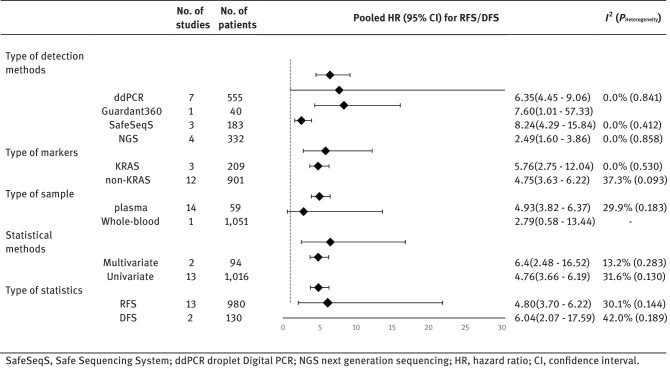

**Table 3:** Subgroup analysis: effect of ctDNA on OS according to the various subgroups is presented

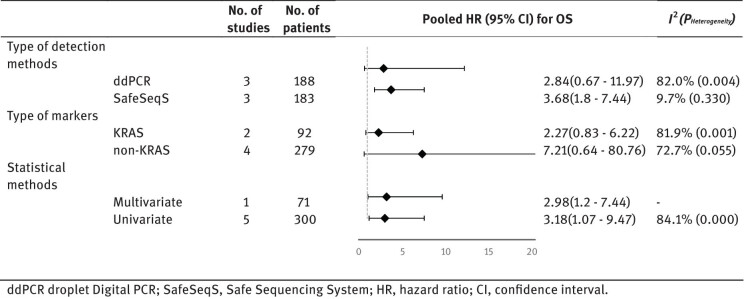

A subgroup analysis of DFS/RFS was conducted according to the type of detection method, marker, sample, statistical method, and statistical tests. Subgroup analysis using type of detection method and statistical methods demonstrated consistent results and that the effect of ctDNA positivity on DFS/RFS remained significant (type of detection method [ddPCR, pooled HR = 6.35, 95% CI: 0.74–1.9, *p* < 0.001; Guardant360, pooled HR = 7.60, 95% CI: 1.01–57.33, *p* < 0.001; Safe-Sequencing, pooled HR = 8.24, 95% CI: 4.29–15.84, *p* < 0.001; NGS, pooled HR = 2.49, 95% CI: 1.60–3.86, *p* < 0.001]; statistical methods [multivariate, pooled HR = 6.4, 95% CI: 2.48–16.52, *p* < 0.001; univariate, pooled HR = 4.76, 95% CI: 3.66–6.19, *p* < 0.001]) and heterogeneity was absent (analysis based on type of detection method [ddPCR, *I* ^2^ = 0.0%, *p* = 0.841; Guardant360, –; Safe-Sequencing, *I* ^2^ = 0.0%, *p* = 0.412; NGS, *I* ^2^ = 0.0%, *p* = 0.858]; and statistical method [multivariate, *I* ^2^ = 13.2%, *p* = 0.283; univariate, *I* ^2^ = 31.6%, *p* = 0.130]). Of the two markers assessed in the subgroup analysis, only one was determined from whole-blood samples, whereas the other was determined from plasma samples, and the findings of the subgroup analysis remained significant (plasma, pooled HR = 4.93, 95% CI: 3.82–6.37, *p* = 0.201; whole blood, pooled HR = 2.79, 95% CI: 0.58–13.44, *p* < 0.001), despite negligible heterogeneity (plasma, *I* ^2^ = 29.9%, *p* = 0.183; whole-blood, –). ctDNA was detected using numerous markers in the included studies, with the exception of Kirsten rat sarcoma virus (KRAS), which was detected using a single marker. Based on the results, the markers were divided into two subgroups: KRAS and non-KRAS (KRAS, pooled HR = 5.76, 95% CI: 2.75–12.04, *p* < 0.001; non-KRAS, pooled HR = 4.75, 95% CI: 3.64–6.22, *p* < 0.001). Insignificant heterogeneity was observed in subgroup analysis of stratified factors for markers and combined findings (KRAS, *I* ^2^ = 0.0%, *p* = 0.530; non-KRAS, *I* ^2^ = 37.3%, *p* = 0.093). We used the same criteria for both RFS and DFS and conducted a subgroup analysis to ensure that the final findings were not influenced by the subgroup analysis. The RFS and DFS groups did not exhibit considerable heterogeneity (RFS, *I* ^2^ = 30.1%, *p* = 0.144; DFS, *I* ^2^ = 42.0%, *p* = 0.189) and the pooled findings were robust (RFS, pooled HR = 4.80, 95% CI: 3.70–6.22, *p* < 0.001; DFS, pooled HR = 6.04, 95% CI: 2.07–17.59, *p* = 0.001).

OS-related subgroup analyses were conducted based on the type of detection method, markers, and statistical methods. The results of the subgroup analysis of the two different assays were as follows: in the subgroup of three studies that used ddPCR as the detection method, the pooled HR was nonsignificant (pooled HR = 2.84, 95% CI 0.67–11.97, *p* = 0.155), and the heterogeneity was high (*I* ^2^ = 82.0%, *p* = 0.004), whereas in the subgroup of the three studies that used Safe-SeqS as the detection method, the pooled HR was significant (pooled HR = 3.68, 95% CI 1.80–7.44, *p* < 0.001), and there was no heterogeneity (*I* ^2^ = 9.7%, *p* = 0.330). None of the combined HRs were significant in the subgroup analysis of marker types (KRAS, pooled HR = 2.27, 95% CI 0.83–6.22, *p* = 0.110; non-KRAS, pooled HR = 7.21, 95% CI 0.64–80.76, *p* = 0.109), and a high heterogeneity was noted (KRAS, *I* ^2^ = 81.9%, *p* = 0.001; non-KRAS, *I* ^2^ = 72.7%, *p* = 0.055). In subgroup analysis based on statistical methods, univariate and multivariate analyses pooled the same results (univariate, pooled HR = 3.18, 95% CI 1.07–9.47, *p* = 0.038; multivariate, pooled HR = 2.98, 95% CI 1.20–7.44, *p* = 0.105), and heterogeneity was significant (univariate, *I* ^2^ = 84.1%, *p* < 0.001; multivariate, –).

### 3.5 Sensitivity analysis and publication bias

**Figure 3:**
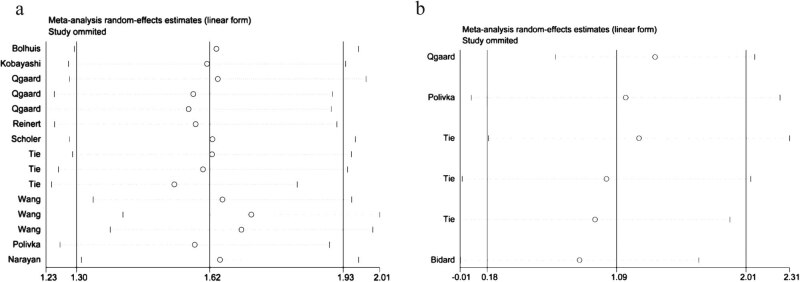
(a) Sensitive analysis about RFS/DFS. (b) Sensitive analysis about OS.

We conducted a sensitivity analysis by calculating and examining the combined HR after random omission of an included study to determine the influence of excluding a single study on the overall result. The outcomes of the sensitivity analysis showed that the overall findings of this research on RFS/DFS were robust (Figure 3a). The study conducted by Øgaard et al. [32] was a source of heterogeneity in the OS-related sensitivity analysis (*I* ^2^ = 32.3%, *p* = 0.206) (Figure 3b). When this study was omitted, the pooled results changed, suggesting that the combined results for OS were unstable.

**Figure 4:**
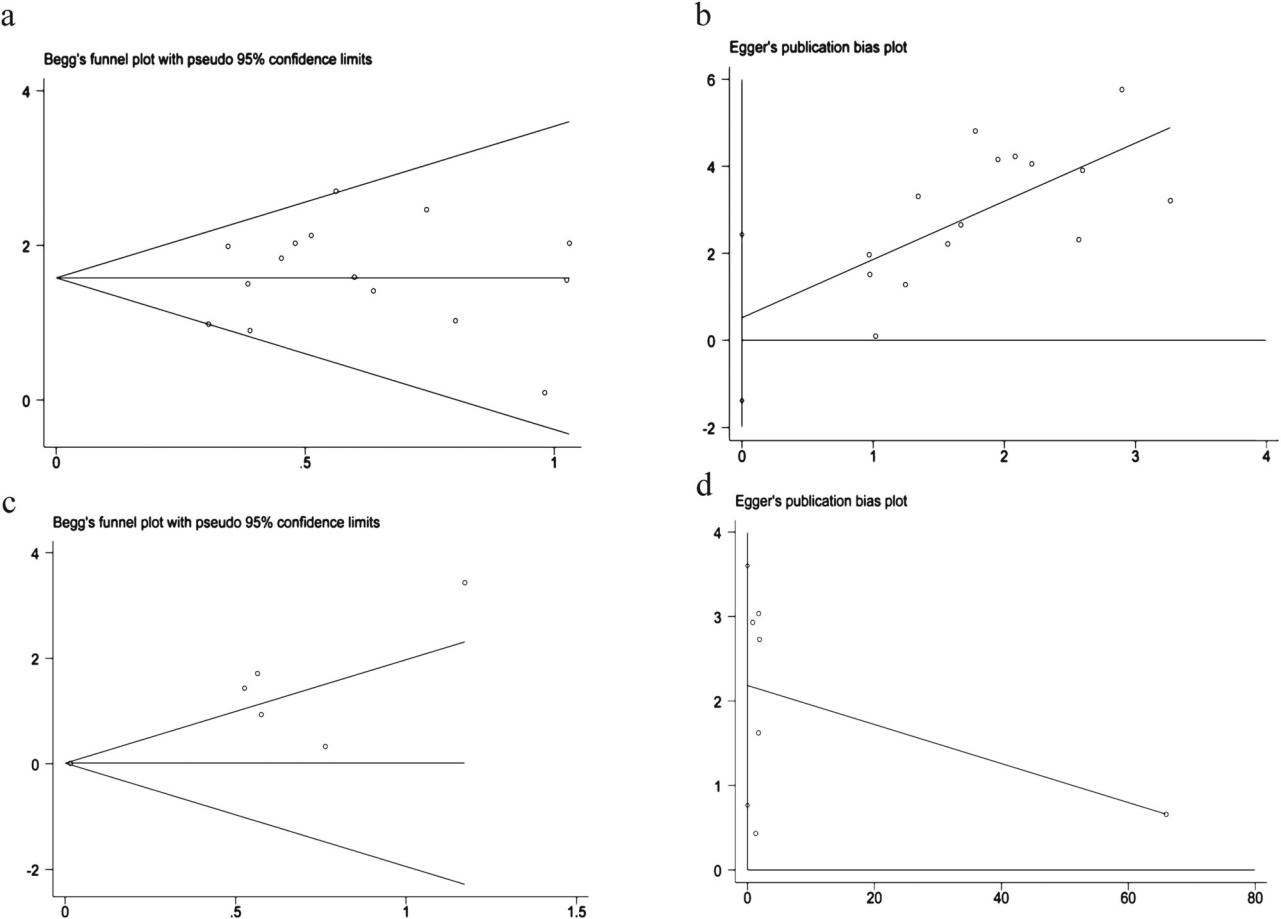
(a and b) Publication bias about RFS/DFS (Begg’s and Egger’s). (c and d) Publication bias about OS (Begg’s and Egger’s).

Begg’s and Egger’s techniques were used to analyze the presence of publication bias in this meta-analysis. The results demonstrated that no publishing bias existed, either qualitatively or quantitatively, in RFS/DFS (Figure 4a and b). However, the results clearly showed the presence of publication bias in OS (Figure 4c and d).

## 4 Discussion

Subgroup, sensitivity, and publication bias analyses were performed for the combined outcomes of OS. The results suggested that the combined outcome of OS was unstable, and therefore, we exercised caution when inferring that the OS rate of ctDNA-positive patients cannot be considered lower than that of ctDNA-negative patients. This may have been due to the small number of studies assessed in this meta-analysis. However, patients who had a ctDNA-positive result had poorer RFS/DFS than those who had a ctDNA-negative result. We conducted a subgroup study of RFS/DFS, considering sample collection time, detection method, detection marker, source of the marker, and statistical meth, and we found that the pooled results for all subgroups were statistically significant.

Samples were collected at baseline, after the operation, postoperative chemotherapy, and treatment completion. Combined data indicated that patients who tested positive for ctDNA had worse DFS/RFS regardless of the period of sample collection. Tumor cellular DNA is released into the bloodstream after surgery, resulting in elevated ctDNA levels, after which ctDNA levels decrease [39]. ctDNA was detected in most of the resectable and unresectable cancer cases, and alterations in ctDNA serve as early indicators of unfavorable disease behavior [25]. After local therapy for metastatic CRC, the presence of ctDNA was associated with an elevated risk of recurrence and a short time to failure [40]. This may be due to the presence of residual cancer cells or metastases [13].

The status analysis of ctDNA may be utilized not only as a predictive predictor of the disease but also for the diagnosis and monitoring of a broad spectrum of malignancies, providing data for accurate treatment choices for patients [23,41,42]. In particular, CRC leads to a certain genetic susceptibility, and in some cases, early diagnosis and prevention can be accomplished by testing patients for the presence of genetic mutations associated with the disease [43]. Serial measurements of ctDNA-related methylation markers in patients with CLM receiving neoadjuvant chemotherapy may be useful to detect early patient responses and can be utilized to monitor patient response [44]. ctDNA might be useful for identifying early anti-epidermal growth factor receptor resistance [45,46,47]. In particular, monitoring KRAS in ctDNA may be useful to assess therapy response and treatment plans [19]. Monitoring KRAS mutations during therapy to identify ctDNA offers new modalities for drug resistance prediction, treatment regimen selection, and drug detection [48]. It was also hypothesized that the plasma ras-mutant allele fraction (MAF) could be used not only to predict the fate of CRC before therapy but also as a predictive biomarker [48,49]. However, when applying ctDNA testing in clinical settings, it is important to consider the biological processes that influence ctDNA shedding from tumors. Most ctDNAs are eliminated or are present below the detection threshold after metastasectomy for oligometastatic lesions that responded well to treatment [21].

Several methods had been developed to detect ctDNA, including real-time or digital PCR, NGS, and BEAMING (beads, emulsions, amplification, and magnetics). Each technique has distinct assay performance characteristics [50]. Our subgroup analysis revealed a low degree of variation in the detection mode. Although ddPCR-based analysis is highly specific and beneficial for mutation monitoring, its sensitivity restricts its use for early cancer identification [51]. NGS-based tests are more sensitive for ctDNA detection and produce only a few errors [18,25]. Further research is required to confirm the influence of various detection technologies on ctDNA diagnosis. However, it should be noted that ctDNA test results may also be affected by other factors, such as sample quality and analytical methods.

ctDNA is synthesized in tumor cells and is then released into circulation by necrosis, apoptosis, and secretion [12,13,16]. These findings are further supported by the results of a study that demonstrated remarkable concordance between blood and tumor samples for determining rat sarcoma (RAS) status and BRAF mutations, especially in patients with CLM [52,53,54,55]. However, various discrepancies in the status of detection markers in plasma and tissues have been reported, and antitumor treatment and metastatic location may be significant variables affecting this discrepancy [56]. The RAS status was more constant in left-sided CRC (91.3%), rectal cancer (83.5%), and right-sided CRC (76.9%) [57]. Additionally, the present study showed that ctDNA has a high sensitivity for CRC detection and could be used as a first-line diagnostic modality by determining the RAS and BRAF status [55]. The increase in MAF enables the early identification of chemoresistance and predated tumor markers and CT [58]. ctDNA produces more accurate results than CEA in detecting disease recurrence and progression earlier and more efficiently than imaging (the current standard biomarker for CRC) [59]. In CLM, ctDNA levels correlated well with CEA levels [22,60]. ctDNA, in combination with circulating tumor cells, may be used to determine the dynamic features of malignancies [17]. Additional studies have shown a strong correlation between ctDNA levels in urine and ascites and ctDNA levels in the blood, establishing new methods for detecting ctDNA [36,61–63].

It is worth noting that our meta-analysis had several limitations. First, the studies were chosen based on their clinical and methodological diversity. Second, given the small number of studies included, there was a possibility of bias due to the scarcity of data. Third, the markers used for identifying ctDNA vary considerably; for example, some studies used the KRAS gene as the only marker, which may have resulted in certain samples reporting false-negative results and skewing the study findings. Fourth, although studies have performed sensitivity analysis and publication bias evaluation, subgroup analysis revealed no evidence of considerable heterogeneity in RFS/DFS. However, unknown confounding variables were not accounted for. Finally, further large-scale, multicenter, prospective clinical studies are needed in the future.

## 5 Conclusion

In summary, the results of our meta-analysis indicated an increased risk of disease recurrence in the ctDNA-positive group compared with that in the ctDNA-negative group, regardless of the timing of sample collection. There was a required relationship between ctDNA positivity and the recurrence of CLM, which was predicted to be a new biomarker for ctDNA survival prognosis. However, further studies including large-sample clinical trials and fundamental research are required to validate the function of ctDNA in CLM and to investigate the mechanism by which ctDNA develops in human cancers.
